# Locally Advanced Breast Cancer – Strategies for Developing Nations

**DOI:** 10.3389/fonc.2015.00089

**Published:** 2015-04-27

**Authors:** Onyinye D. Balogun, Silvia C. Formenti

**Affiliations:** ^1^Department of Radiation Oncology and Surgery, New York University Langone Medical Center, New York University School of Medicine, New York, NY, USA

**Keywords:** breast cancer, global health, cost-effectiveness analysis, low-income countries, middle-income countries, radiation therapy, chemotherapy

Worldwide, cancer incidence and cancer-related deaths are steadily rising. According to the International Agency for Research on Cancer, new cancer cases rose from 12.7 million in 2008 to 14.1 million in 2012 (1). Similarly, 7.6 million cancer-related deaths occurred in 2008 compared to 8.2 million in 2012. A significant proportion of these cases are attributed to breast cancer, the predominant malignancy affecting women worldwide. Since 2008, breast cancer incidence has increased by over 20% and breast cancer deaths have risen by 14% (1). Although the incidence of breast cancer is still highest in developed countries, women in developing nations are disproportionately dying as a result of this disease. Six of the 10 countries with the highest breast cancer mortality rate are low- to middle-income countries (LMICs) (Figure 1). Moreover, breast cancer in LMICs often presents when locally advanced breast cancer (LABC) (2–4) that can be easily appreciated at physical exam but is still limited to the breast and draining lymph nodes, without clinical evidence of metastatic spread. LABC is defined as tumors: (1) more than 5 cm in diameter, (2) involve the skin or the underlying pectoral muscles, (3) involve axillary, supraclavicular, and/or infraclavicular lymph nodes, or (4) inflammatory breast cancer. Despite being confined to the breast and regional nodes, locally advanced stage often heralds the rapid onset of metastatic disease, explaining high mortality rates. Solutions are needed to address this health issue. We propose practical strategies to improve the early detection of breast cancer and the treatment of LABC within developing nations.

**Figure 1:**
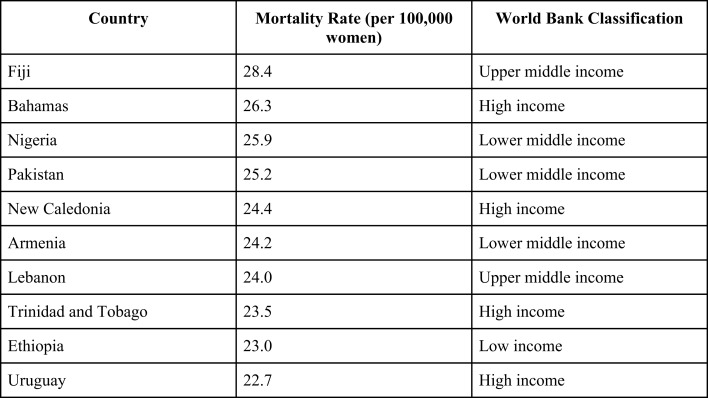
**Age-standardized mortality rates, number of deaths per 100,000 women (1)**.

## Detection

In developed countries, national screening programs have been widely implemented. Although there are tangible benefits to mammographic screening, following the same paradigm in developing nations may not be ideal or feasible. First, women in several developing nations are diagnosed at a younger age than their counterparts in developed countries. In the United States, the median age at diagnosis is 61 years old. In comparison, the median age at diagnosis is 50 years old among women in Mexico (5) and 46 years old among Egyptian women (6). The sensitivity of mammography is affected by several factors including age and breast tissue density. In women <50 years old, the sensitivity of mammography can be as low as 68% (7). Digital mammography improves the detection of cancer in younger women but is associated with higher costs compared to film mammography. In a study of over 40,000 women, the accuracy of digital mammography was significantly higher than that of film mammography for women under 50 years old, pre- and peri-menopausal women and those with heterogeneously dense or extremely dense breasts on mammography (8). Screening mammograms are performed in women without symptoms of breast cancer. Diagnostic mammograms are used to diagnose breast cancer once suspicious findings have been noted on screening mammogram or if an individual has symptoms suggestive of breast cancer. Diagnostic mammograms involve more views of the breast and take longer to perform. In addition, a radiologist is present to immediately interpret the exam. When used for screening or diagnostic purposes, digital mammograms cost $11 or $33 more per examination, respectively (9). Restricting the use of digital mammograms to women under 50 years, those most likely to benefit from a more accurate assessment of breast densities, would still prove too expensive for low- to middle-income nations. According to the World Health Organization, a cost-effective health intervention is one to three times a country’s gross domestic product (GDP) per capita. Age-targeted digital mammography would cost $26,500 per quality-adjusted life year (QALY) (10), well above the cost-effective threshold for most LMICs.

For developing nations, screening mammography programs are likely cost-prohibitive with questionable benefits. This is especially true in populations with a significant number of young breast cancer patients, for whom mammography is less likely to detect malignancies and leads to more false-positive results (11–13). It would be unwise for nations with limited resources to indiscriminately adopt the same screening strategy. Financial resources are likely better invested in public awareness campaigns and training community health workers to educate the public and perform clinical breast exams (CBE) (2, 14, 15). For example, a cost-effectiveness analysis of breast cancer interventions in Ghana revealed that mammographic screening of women 40–69 years old would cost $12,908 per disability adjusted life year (DALY) averted. In contrast, biennial CBE and mass media awareness campaigns would cost $1299 and $1364 per DALY averted, respectively (16). Distrust of the medical system and myths about breast cancer persist, leading women to rely on traditional healers in lieu of health centers to their detriment (17, 18). These issues highlight a critical need to invest in education.

## Multimodality Care

Generally, only ~15% of breast cancer patients in LMICs present with Stage I breast cancer and 20–40% present with Stage II disease (19). In sub-Saharan Africa, 40–90% of women present with Stage III–IV disease (20). The same is true for low- to middle-income Latin American countries. In Colombia, 68.2% of patients present with locally advanced disease and in Peru and Mexico, approximately 50% of patients present with advanced disease (21). Although the 3-year survival rate for Stage III patients in high-income countries ranges from 70 to 85%, the survival rate for patients with comparable stage of disease is much lower in developing nations. Optimizing treatment in this subpopulation is part of a reasonable strategy to improve breast cancer mortality in developing countries.

### Surgery

Surgery plays an important role in the management of LABC. In developing countries, modified radical mastectomy (MRM) continues to be the mainstay of surgical treatment. In Yemen, approximately 50% of women undergo MRM and an additional 10% undergo radical mastectomy (22). Unfortunately, surgical techniques for mastectomies are sometimes suboptimal. In USA and the United Kingdom, most breast surgeons have undergone surgical oncology fellowships. In contrast, opportunities for specialty training are limited in LMICs. Moreover, quality control protocols and data regarding mastectomies in developing countries, including the rate of negative margins and the number of lymph nodes excised, are lacking (23). Studies are needed to assess the quality of mastectomies and pinpoint areas for improvement that can lead to better outcomes.

Fear of deformity is among the multiple concerns that breast cancer patients face during treatment (24). Several studies demonstrated that body image is superior in women who undergo breast conservation therapy (BCT) or mastectomy with reconstruction rather than those who have undergone mastectomy without reconstruction. Interestingly, overall quality of life is the same for patients whether they undergo mastectomy with or without reconstruction, suggesting that satisfaction with body image is only one component of global quality of life after breast cancer (25). Although providing opportunities for reconstruction would be ideal, this should be a lower priority goal in a limited resource setting, especially since this procedure can cost between $15,000 and $50,000.

### Chemotherapy

Neoadjuvant chemotherapy is recommended for women with LABC. In some cases, neoadjuvant chemotherapy can significantly shrink the tumor making lumpectomy possible. It is essential that developing nations implement cost-effective chemotherapeutic regimens. The WHO Model List of Essential Medicines presents a core list of the minimum medicine needs for a healthcare system. In addition, it denotes essential medicines for diseases like cancer that require specialized care. Among the 30 cytotoxic and anti-hormonal therapies, the breast cancer-related agents include carboplatin, cyclophosphamide, docetaxel, doxorubicin, fluorouracil, methotrexate, paclitaxel, and tamoxifen. Provision of these agents may be a realistic target for upper-middle-income nations. However, LMICs may be best served by focusing on access to three to four of these medications. We propose paclitaxel, doxorubicin, cyclophosphamide, and tamoxifen as the basic chemotherapeutic elements of breast cancer care. Chemotherapy recommendations according to national resources have also been published by the Breast Health Global Initiative (26).

The Academic Model Providing Access to Healthcare (AMPATH) is a successful model of chemotherapy delivery in Kenya, a low-income nation (27). AMPATH is a collaboration between Moi University School of Medicine in Kenya and North American academic medical centers. Since 2005, cancer care services have been available and breast cancer represents over 60% of female-specific malignancies. The AMPATH Oncology Pharmacy Service (AOPS) stocks doxorubicin, cyclophosphamide, and tamoxifen in addition to 15 other chemotherapy-related agents. AC chemotherapy appears to be the most readily available for women in developing nations. Nearly 50% of patients receiving neoadjuvant chemotherapy in Ibadan, Nigeria were treated with doxorubicin and cyclophosphamide (3). The AOPS experience also provides other insights for LMICs regarding issues of cost containment, personnel training, disposal, preparation/dispensing, and storage associated with chemotherapy. For instance, by centralizing inventory and monitoring monthly use statistics, AOPS minimized the risk of drug shortages and negotiated better prices. The latter is especially important because many patients are uninsured and must bear the total out-of-pocket costs. Often, patients cannot afford chemotherapy and will forego this aspect of treatment. Ntirenganya et al. reported that 35% of women with breast masses in Sierra Leone did not seek medical care due to lack of money (18). By making chemotherapy more affordable, healthcare institutions can ensure that patients are more likely to receive optimal care thereby improving cancer outcomes. It will also be necessary to invest in supportive therapies such as antiemetics for successful implementation of chemotherapy.

Another cost-effective strategy is to combine oophorectomy and hormonal therapy. In a study of 709 premenopausal Vietnamese and Chinese women with Stage IIA–IIIA breast cancer, patients were randomized to undergo oophorectomy at the time of mastectomy and adjuvant tamoxifen versus receiving this combined hormonal treatment at recurrence (28). At 5 years, oophorectomy and tamoxifen up front led to a statistically significant disease-free and overall survival benefit. Moreover, this intervention cost $350 per year of life saved.

Targeted agents, such as trastuzumab, are noticeably absent from the WHO Model List of Essential Medicines and likely the pharmacies of most developing nations. Assessments in Peru, Costa Rica, and Mexico demonstrate that providing trastuzumab will cost over $10,000 per DALY and is consequently not recommended (29, 30). Therefore, unfortunately HER2-directed therapies should not be a priority for low- to middle-income nations.

### Radiation therapy

Radiation therapy is an important component of care for women with LABC. Several randomized trials have demonstrated the local recurrence and mortality benefit associated with adjuvant radiation therapy after mastectomy (31). Unfortunately, radiation therapy services are severely lacking in LMICs. Of 139 LMICs, 55 (39.5%) have no radiation therapy facilities (32) and 29 of these are African nations (33). In most high-income countries, at least one radiotherapy machine is available for every 250,000 people. In contrast, in nearly 20 LMICs, only one machine is available for over 5 million people. Ideally, LMICs should invest in establishing radiation therapy infrastructure and training personnel. However, decision-analytic models estimate that post-mastectomy radiation therapy costs $12,000–$22,600 per QALY (34, 35). Although this is cost-effective for most upper-middle-income countries, it is unlikely to be sustainable for low to lower-middle-income countries. Innovative methods are needed to provide radiation therapy at lower cost in these developing nations. One strategy may be to shorten the course of radiation therapy. Hypofractionated breast radiotherapy is commonly used after lumpectomy. Although decreasing the total dose may enhance the therapeutic ratio, previous studies suggest that 3 Gy per fraction post-mastectomy is associated with unacceptable brachial plexus toxicity (36). Additional studies are needed to identify hypofractionated radiation therapy regimens that can safely treat both the chest wall and regional lymph nodes.

Concurrent chemoradiation therapy may also shorten the overall length and cost of treatment while maintaining treatment efficacy. Among 105 women treated with neoadjuvant concurrent paclitaxel and radiotherapy to the breast and regional nodes, 34% achieved a pathological response including over 50% of triple-negative patients (37). Shortening chemotherapy and radiation therapy courses also makes treatment more convenient to patients, since patients in LMICs often have to travel long distances and temporarily live far away from their homes to undergo treatment.

Finally, simplifying the radiation therapy planning process can reduce the technical fees and overall cost of radiation therapy. Zhao et al. published their algorithm for determining the optimal placement of tangential beams (38). This method does not require manual beam placement by physicians, a time-saving feature especially in developing countries with a limited number of physicians. Similar methods for designing regional lymph node radiotherapy fields are needed.

## Conclusion

Locally advanced breast cancer contributes significantly to cancer mortality among women worldwide. It is particularly important to address this disease in developing nations, where over 70% of all cancer cases will occur by 2020. There is an overwhelming need for systematic studies that pinpoint areas of need within the context of each developing nation and also within regions in a developing nation. Research in these settings and dissemination of these data (39) will guide the judicious use of available financial and human resources. In this article, we have suggested strategies for addressing LABC in LMICs. Potential solutions include (1) investing in CBE and awareness campaigns, (2) gathering data and establishing quality control protocols for mastectomies, (3) focusing on the provision of few but effective chemotherapeutic agents, and (4) investigating cost reduction methods for radiation therapy including shorter regimens.

## Conflict of Interest Statement

The authors declare that the research was conducted in the absence of any commercial or financial relationships that could be construed as a potential conflict of interest. The Associate Editor Daniel Grant Petereit declares that, despite having collaborated with author Silvia C. Formenti, the review process was handled objectively and no conflict of interest exists.
